# Genetic diversity of avocado (*Persea americana* Mill.) germplasm using pooled sequencing

**DOI:** 10.1186/s12864-019-5672-7

**Published:** 2019-05-15

**Authors:** Mor Rubinstein, Ravit Eshed, Ada Rozen, Tali Zviran, David N. Kuhn, Vered Irihimovitch, Amir Sherman, Ron Ophir

**Affiliations:** 1 Department of Fruit Trees Sciences, Institute of Plant Sciences, Agricultural Research Organization, Volcani Center, Rishon Lezion, Israel; 2 Subtropical Horticulture Research Station, United States Department of Agriculture—Agriculture Research Service, Miami, FL USA

**Keywords:** F_ST_, Kinship, Germplasm collection, Avocado, SNP

## Abstract

**Background:**

Discovering a genome-wide set of avocado (*Persea americana* Mill.) single nucleotide polymorphisms and characterizing the diversity of germplasm collection is a powerful tool for breeding. However, discovery is a costly process, due to loss of loci that are proven to be non-informative when genotyping the germplasm.

**Results:**

Our study on a collection of 100 accessions comprised the three race types, Guatemalan, Mexican, and West Indian. To increase the chances of discovering polymorphic loci, three pools of genomic DNA, one from each race, were sequenced and the reads were aligned to a reference transcriptome. In total, 507,917 polymorphic loci were identified in the entire collection. Of these, 345,617 were observed in all three pools, 117,692 in two pools, 44,552 in one of the pools, and only 56 (0.0001%) were homozygous in the three pools but for different alleles. The polymorphic loci were validated using 192 randomly selected SNPs by genotyping the accessions within each pool. The sensitivity of polymorphic locus prediction ranged from 0.77 to 0.94. The correlation between the allele frequency estimated from the pooled sequences and actual allele frequency from genotype calling of individual accessions was r = 0.8. A subset of 109 SNPs were then used to evaluate the genetic relationships among avocado accessions and the genetic diversity of the collection. The three races were distinctly clustered by projecting the genetic variation on a PCA plot. As expected, by estimating the kinship coefficient for all the accessions, many of the cultivars from the California breeding program were closely related to each other, especially, the Hass-like ones. The green-skin avocados, e.g., ‘Bacon’, ‘Zutano’, ‘Ettinger’ and ‘Fuerte’ were also closely related to each other.

**Conclusions:**

A framework for SNP discovery and genetically characterizing of a breeder‘s accessions was described. Sequencing pools of gDNA is a cost-effective approach to create a genome-wide stock of polymorphic loci for a breeding program. Reassessing the botanical and the genetic knowledge about the germplasm accessions is valuable for future breeding. Kinship analysis may be used as a first step in finding a parental candidates in a parentage analyses.

**Electronic supplementary material:**

The online version of this article (10.1186/s12864-019-5672-7) contains supplementary material, which is available to authorized users.

## Background

Avocado (*Persea americana* Mill.) is a subtropical tree which belongs to the *Lauraceae* family, one of the oldest known flowering plant families [1]. Its natural habitat is Central and South America. The *P. americana* species is conventionally classified as Guatemalan (*P.americana* var. *guatemalensis* L.), Mexican (*P.americana* var. *drymifolia*) and West Indian (*P.americana* var. *americana* Mill). The three races are distinguished by their traits: the Mexican avocados are tolerant to cold and have high oil content; the Guatemalan are less cold tolerant, and the West Indian types are tolerant to salty soil but sensitive to cold [2]. Other differences found in fruit and seed size, skin thickness and surface, oil content of the fresh fruit, maturity season, and anise-scented leaves, refine their horticultural classification [3] . Commercial cultivars are considered to be hybrids of the three races, mainly crosses between the Guatemalan and the Mexican types [4]. The division to races has been supported by genetic markers such as restriction fragment length polymorphisms (RFLP) [5], random amplified polymorphic DNA (RAPD) [6], and microsatellite markers [4, 7, 8]. In light of the high-throughput technologies that have been developed in the last decade such as microarray, next-generation sequencing (NGS), and successively genotype-by-sequencing (GBS), single-nucleotide polymorphisms (SNPs) have emerged as a useful genetic marker tool to estimate variability on a genome-wide scale [9–12]. A ‘good’ or informative marker is a polymorphic marker in the population under study that varies due to the bi-parental cross. Therefore, compiling a stock of SNPs that is polymorphic in any segregating population is difficult. To increase the chances of marker segregation in any future crossbreeding from the germplasm collection, SNPs with equal proportions of the SNP alleles throughout the collection should be compiled. A cost-effective approach to assessing allele frequency in a germplasm collection is sequencing a pool of the individuals’ DNA [13–15]. Previous studies have suggested a high accuracy of allele frequency determined from sequencing DNA pools [16–18]. Exome-wide SNP discovery can reduce the genome’s complexity and increase the sequence-read coverage [15]. Moreover, this approach increases the chances of finding markers close to causative trait loci. However, estimating the allele frequency directly from expression data might lead to a biased result or even false loss-of-heterozygosity (LOH) deductions due to allele-specific expression.

In this report, we suggest a framework for SNP discovery and genetic characterization of a germplasm collection based on sequencing of genomic DNA (gDNA) pools. The genomic complexity in the SNP discovery was reduced by aligning the sequencing reads with the avocado reference transcriptome. Genotyping avocado accessions from the germplasm collection using a randomly selected subset of loci illustrated the sensitivity of the pools to predicted polymorphic loci. Furthermore, using this subset of SNPs, we suggested an alternative approach to characterize the genetic diversity of a crop germplasm. Instead of using either botanical characters or genetic markers, we used the consensus of both to define three core sets of accessions from the germplasm collection representing the three races. Finally, we assigned the rest of the accessions to either one of the races or to an admixed group and delineated the genetic structure of the collection.

## Results

### SNP discovery by sequencing of DNA pools

SNPs were discovered by sequencing (Illumina HiSeq-2000) of three gDNA pools of the Israeli avocado germplasm bank (IAGB) and aligning them to avocado mRNA transcriptome. The rationale behind this approach was supported by two arguments. First, for SNP discovery, gDNA is favored over mRNA to avoid false calls that might be a result of allelic specific expression. Second, sequencing the entire collection using a pool of DNA as opposed to individual samples is cost effective. As mentioned hereinbefore, previous studies suggested that the three races, as well as their hybrids, are distinguishable by genetic markers [5–7]. Thus, to ensure that both alleles of a SNP will be presented in all races, we divided the collection into three samples that were pooled by race type, i.e., Guatemalan, Mexican, and West Indian. In total, 507,303 loci were presented by both alleles. Of these, only 345,617 (68%) were polymorphic in each of the three pools (Table 1). There were 117,692 (23.2%) loci, which were polymorphic in two pools. The profile of polymorphic loci in Guatemalan pool, in Mexican pool, and non-polymorphic in West Indian pool (pG-pM-npWI) was significantly more frequent 48% (χ^2^-test, df = 2, *p*-value< 0.001) than the two other profiles (pG-pWI-npM [39%]; pWI-pM-npG [13%]). There were 44,552 (8.8%) polymorphic loci in only one of the pools and 56 (0.0001%) loci, which included the two alleles but were non-polymorphic in all the pools. West Indian pool was the most polymorphic (36%; χ^2^-test, df = 2, *p*-value< 0.001) as the profile (pWI-npM-npG) was most frequent in single-polymorphic-pool loci. The loci percentage of Guatemalan and Mexican pool in this profile category was 34 and 30% respectively.

**Table 1:** Distribution of polymorphism in pools

Number of polymorphic pools	Counts	Percentage of total
Three	345,617	68.0%
Two	117,692	23.2%
One	44,552	8.8%
None	56	0.0001%
Total	507,917	100.0%

The prediction of the polymorphic loci was validated by random sampling of 192 loci and calling accession genotypes using the Fluidigm platform. The loci distribution comprised 94 loci that were polymorphic in all three pools (Guatemalan, Mexican, and West Indian), 72 loci that were polymorphic in two out of the three pools, and 22 were polymorphic in only one of the pools. Only four loci were polymorphic collectively but not within the pools. In other words, the pools were typed as either allele A or allele B for those four loci, yet both alleles were represented in the genotyped accessions. A comparison of the 192 loci predicted polymorphic calls from the sequencing pools and the actual calls showed a good predictive power. The true positive percentage values of polymorphic loci were in a range of 83 to 86% for the three pools (Table 2; AB calls). For the Guatemalan, Mexican, and West Indian pools, the sensitivity of the prediction (i.e., the true positive rate) of polymorphism was estimated as 0.94, 0.86, and 0.77 respectively. The correlation coefficient (r) of allele proportions estimated from the sequenced pools and those from genotyping of the collection was 0.8 (Additional file 5; Figure S2).

**Table 2:** Comparison of polymorphic status of loci in the sequenced pools to actual polymorphism in the collection

Pool												
	Guatemalan				Mexican				West Indian			
Accessions	AA	AB	BB	*NA*	AA	AB	BB	*NA*	AA	AB	BB	*NA*
AA	1	13	1	1	2	14	0	12	1	9	0	1
AB	1	151	0	9	7	130	6	9	15	126	18	4
BB	0	1	0	3	0	1	6	3	0	3	1	3
*NA*	0	11	0	0	0	11	0	1	1	9	1	0
True positive percentage	50%	86%	0%	0%	22%	83%	50%	4%	6%	86%	5%	0%

NA – missing data

### Germplasm collection genetic structure

**Fig. 1:**
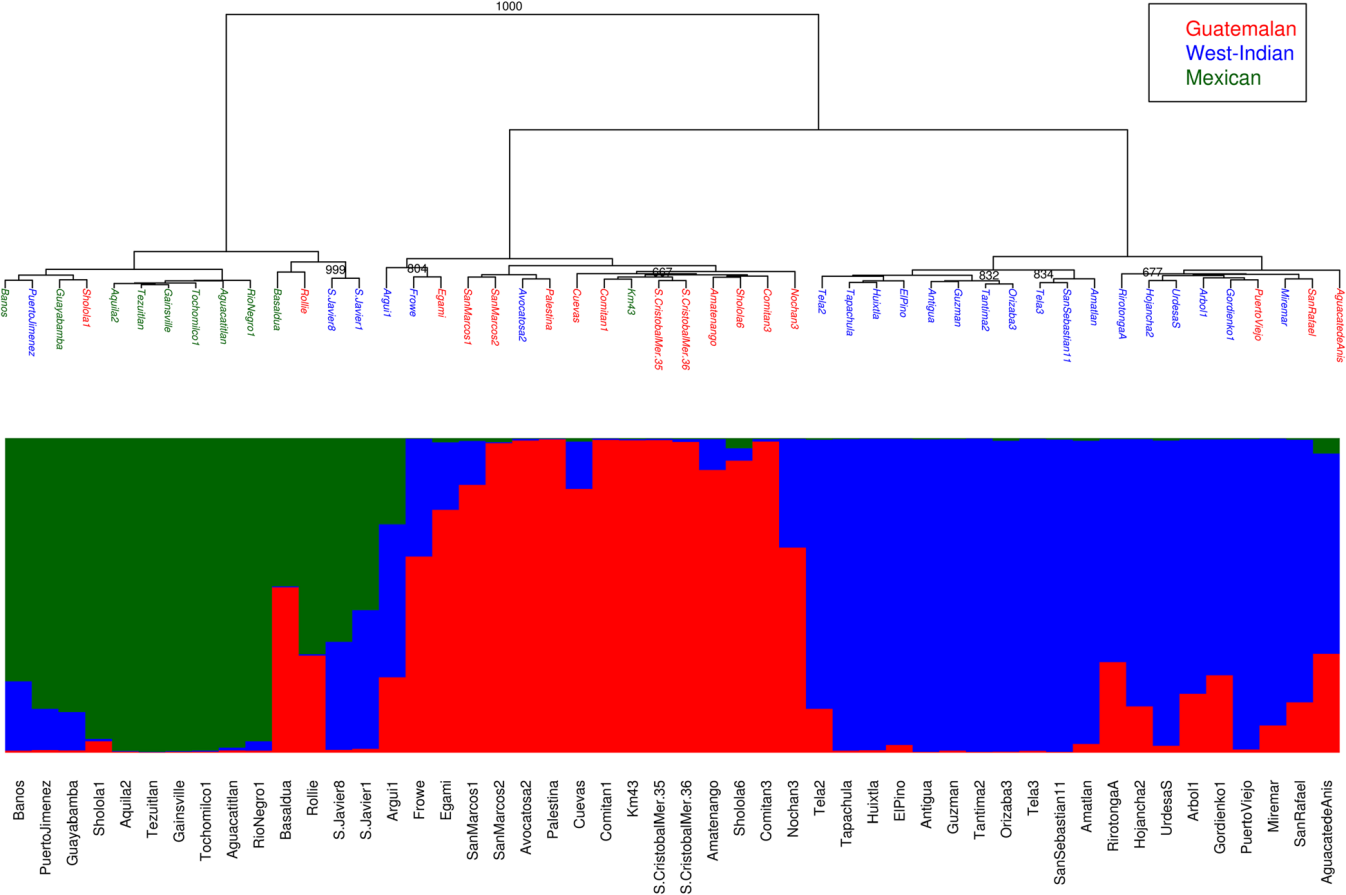
Genetic relationship dendrogram and genetic structure of germplasm collection. Accession names on the dendrogram node-leaves are colored according to morphological characteristics distinct to the three races. Clustering of genetic groups was based on Ward’s method and the distance method of 1-proportion of shared alleles (PSA). Only bootstrap values > 650 are presented with a confidence of the bootstrap-value out of 1000. Genomic admixture was estimated by STRUCTURE program with three subpopulations (K = 3). The average result over 20 simulations was calculated using CLUMPAK, and plotted as a color coded bar plot

The genetic structure and relationships among IAGB accessions were assessed using the polymorphic loci validation subset. Markers were filtered out based on minor-allele frequency, linkage equilibrium, and call failure criteria (see methods) leaving 109 SNPs. The genetic relationships of the IAGB accessions were depicted by a dendrogram, which splits them into three distinct clusters (Fig. 1). Those clusters were roughly correlated with both the morphological and the genetic structure of the avocado accessions (Fig. 1). Only 53% of the Guatemalan, 70% of the Mexican, and 61% of the West Indian accessions in the pools showed congruent race classification in all three methods. The incongruence between the three methods raises some doubts as to whether any of them can be used to identify the race type of avocado. Thus, we selected accessions that were assigned to the same race based on the three methods and were therefore most genetically and morphologically representative of the race to which they were assigned. These accessions were defined as three core sets, one for each race, and were expected to contain the most divergent sets of accessions as they are not yet admixed as a result of breeding. To support this argument, the degree of divergence of the three groups of accessions that were defined by each one of the methods or by a combination of those methods was estimated by F_ST_ (Table 3). The Bayesian cluster method, STRUCTURE, estimated the genetic background of the core-set accessions as homogeneous and therefore highly divergent. However, the median F_ST_ of the core sets was the largest (Table 3), suggesting that the core sets are the most representative of their race type. We therefore used them as a reference to reclassify the rest of the accessions in the collection.

**Table 3:** Classification of races by different methods

Classifying method	# Mexican	# Guatemalan	# West Indian	Median of F_ST_
A – Botanical characterization	10	17	23	0.23
B – PSA dendrogram	14	16	20	0.36
C - STRUCTURE (Q > 0.85)	9	11	16	0.65
A & B	9	12	17	0.56
A & C	7	9	14	0.41
B & C	9	11	16	0.65
A & B & C	7	9	14	0.73

F_ST_ – fixation index

Based on the core sets, we reclassified the accessions that were incongruent within the 50 accessions that were botanically classified as Mexican, Guatemalan, and West Indian [19] as well as the rest of the avocado accessions including the unidentified accessions and the commercial cultivars. The classification was performed by STRUCTURE analysis using the core sets of each race as the reference population for calculating a prior allele frequency distribution. As a result, each accession in the collection was assigned to either one of the core races or as an admixed of two or three races (Additional file 2: Table S2). The number of accessions assigned as Mexican, Guatemalan, and West Indian was 3, 17 and 12 respectively. Cultivar accessions were classified as either hybrids of Guatemalan x Mexican or as Guatemalan. Most of the hybrid accessions (38) were assigned as either Guatemalan x West Indian of which mostly unidentified or as Guatemalan x Mexican of which mostly known commercial cultivars (e.g., ‘Pinkerton’, ‘Ettinger’, and ‘Fuerte’). Interestingly, ‘Hass’ which was previously described as Mexican x Guatemalan hybrid [4–6] but mostly Guatemalan [3] was assigned here as 99% Guatemalan. The smallest group was of two accessions (‘Argui 1’, ‘M. Pedro 2’) of complex hybrid (Mexican x Guatemalan x West Indian), which their genome comprises of three races. Two of the accessions that known as Mexican were turned out to be admixed with Guatemalan (‘Basaldua’) and with West Indian (‘Banos’). One accession, which was misclassified as Mexican (‘Km 43’), was found to be mostly Guatemalan. Note that the ‘Km 43’ was clustered together with the Guatemalan avocados in the genetic-relationship dendrogram.

### Avocado accessions’ kinship

**Fig. 2:**
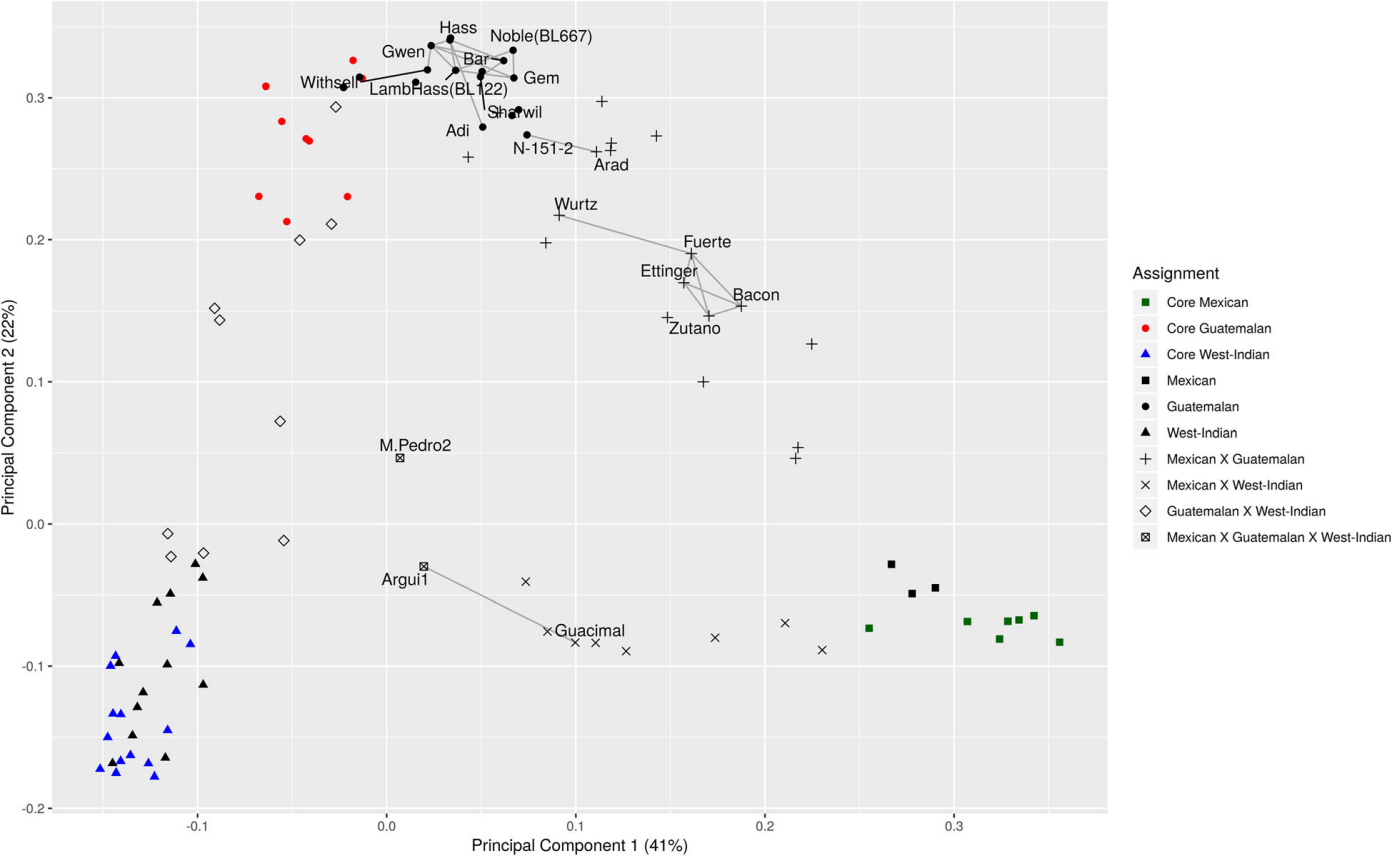
Principal component analysis (PCA) and the second-degree relatedness in avocado germplasm collection. PCA based on identity by state (IBS) of the core sets was performed using the SNPRelate R-package. The rest of the accessions were projected on the PCA space. Additionally, identity by descent (IBD) analysis were performed to detect the level of relatedness. Accessions related to each other with a kinship coefficient ≥ 0.25 are connected by edges

To recapitulate the STRUCTURE clustering and to validate our avocado-accession classifications we then projected 63% of the genetic variation of the IAGB collection on a PCA plot (Fig. 2). The core accessions of each of the three races were most distant from each other. The bi-racial hybrids were spread between the original races creating a triangle. The two accessions of the complex (tri-racial) hybrids were closer to the center of the triangle in the PCA space. One of them, ‘M. Pedro 2’ was a slightly closer to the center although according to the classification both are complex types. Surprisingly, most (15 out of 17) of the assigned Guatemalan accessions created a cluster that was slightly separate from the core Guatemalan accessions and they were comprised mostly (13 accessions) of commercial cultivars.

Identity by descent (IBD) analysis to estimate accessions relatedness was performed. In total, 12 accessions were closely related to each other to a second degree. Of these, eight were Guatemalan commercial cultivars, five were Mexican x Guatemalan commercial cultivar hybrids, one was Guatemalan x West Indian hybrid, and one was a complex hybrid. Two groups of related accessions were created. One among the Guatemalan commercial cultivars and the other among the Mexican x Guatemalan commercial hybrids. Moreover, the Guatemalan x West Indian hybrid was closely related to the complex hybrid ‘Argui1’ and the Guatemalan ‘N-151-2’ was related to Mexican x Guatemalan ‘Arad’. Such close relationships may indicate the source of the genome in the hybrids. For example, the Guatemalan avocado ‘N-151-2’ might be the potential contributor of the Guatemalan genomic block, and therefore the Guatemalan-associated traits, to the Mexican x Guatemalan genome of ‘Arad’ as a second-degree relative. The Mexican x West Indian (‘Guacimal’) is related at a second-degree level to the complex (tri-racial) avocado accession ‘Argui 1’. Thus, ‘Argui 1’ other parents, must come from accessions that contain Guatemalan haplotype block.

## Discussion

For many crops, the germplasm is split into sub-species groups based on their original habitat. Generally, those groups have typical traits. During the process of crop development, one might want to transfer a trait that is typical of a specific sub-group to another. Naturally, such introgression can be guaranteed by choosing a pure sub-species (races) accession rather than an admixed one. Identification of a typical sub-species accession based on morphological characteristics only could be misleading. As a result of such a misidentification the sub-species typical traits may not be inherited due to admixture. In avocado, for instance, three sub-species groups exist, which are conventionally notated as races. Several different methods can be used to characterize and classify the different accessions of a germplasm [3, 20, 21]. This study proposed a framework for evaluating the genetic diversity of a germplasm collection based on a selected core set of accessions, which are most representative of the sub-species group. This method is based upon cost-effective and efficient SNP discovery. The relatedness among the IAGB accessions was then estimated by calculating a kinship coefficient.

Sequencing of DNA pools of natural populations was suggested as a cost-effective and reliable approach for estimating the allele frequency [16, 18]. We utilized the pooled-DNA sequencing approach for SNP discovery, and to create a repository of markers for future use. Pooling the entire germplasm collection is the most cost-effective. Our strategy, which relied on known genetic divergence among the three avocado races, divided the collection into three racial pools to maximize the chances for polymorphic-locus discovery. The use of sub-pooling was further supported by the fact that 32.5% of the SNPs were non-polymorphic in at least one of the races. Thus in the case of genome-wide association analysis, an issue of stratification might have been raised, especially for the Mexican race that showed the highest number of non-polymorphic loci. Marker-assisted breeding can benefit from analyzing the polymorphism profile along the three pools. For example, in a case of designing a cross between an accession from the Mexican pool with an accession from the Guatemalan pool. Supposing that markers, which are heterozygous in the former and homozygous in the latter, are required. The genotyping should focus on loci that were polymorphic in the Mexican pool and non-polymorphic in the Guatemalan pool. These loci were described, hereinbefore, as npG-pM-pWI or as npG-pM-npWI. Likewise, any other design can benefit from narrowing the number of putative polymorphic loci by knowing of polymorphism profiles. The sub-pools were chosen according to their botanical characteristics. Yet by genotyping, it was revealed that at least 30% of the accessions in each pool were admixed with either of the other races. This fact implies that the reduction in polymorphism by 32.5% is not only due the divergence of races. Therefore, further study should be conducted to investigate the option of random sub-pooling and how it could contribute to the enrichment of polymorphic loci.

The sequenced pools, in this study, were used mainly for SNP discovery, i.e., estimation of the existence of the SNP alleles rather than estimation of the alleles’ frequency. The high percentage of true positive polymorphic loci indicated that the approach was sensitive to the discovery of SNPs. The Mexican pool showed the lowest true positive rate probably because it was the smallest pool. The correlation of allele frequency between the pooled sequences and the genotype calls of the individual accessions in the pool was high. We expected that this correlation would have been higher [16, 18] if the pools had been comprised of a higher number of accessions [15, 18].

The core sets, in this study, were clearly separated from each other by a principal component dimensions and the hybrid accessions were scattered between their related cores. This suggests that using a combination of morphological characteristics and genetic markers as a reference for classification is a good approach. Interestingly, the Guatemalan core accessions were slightly separated from the assigned Guatemalan accessions in comparison to the other two races whose core and the corresponding assigned accessions were clustered together. The fact that most of the assigned Guatemalan accessions were related to each other might explain their separation from the others. These accessions were mostly comprised of commercial cultivars, suggesting the desirability of Guatemalan traits in the market [2]. Of special interest were the connected accessions on the PCA plot. For instance, three Hass-like cultivars: ‘Gem’, ‘Noble (BL667)’, ‘Lamb-Hass (BL112)’, all products of the California breeding program, were related to each other and to the ‘Hass’ cultivar. Knowing that these Hass-like cultivars, originated from either an open pollination of a ‘Gwen’ seedling (a commercial cultivar resulting of ‘Hass’ x ‘Thille’ cross), or a seedling that originated from ‘Gwen’ x ‘Thille’ cross (a lesser known variety originating from ‘Hass’ seedling) [22], these results were not surprising.

Another interesting feature, emerging from the PCA plot and IBD analysis, was the tight sibling relationships established between four green-skinned cultivars including: ‘Bacon’, ‘Zutano’, ‘Ettinger’ and ‘Fuerte’ (all representing Mexcian x Guatamalan hybrids). ‘Fuerte’ was found as a dooryard seedling in Mexico, and eventually became an important cultivar [7, 22]. ‘Bacon’ and ‘Zutano’, on the other hand, originated in the late 1920s, as seedling trees in Beuna Park, and Fallbrook, California, respectively [22], whereas, ‘Ettinger’ was bred from an open pollinated ‘Fuerte’ seedling, in Kfar Malal, Israel, in the late 1930s as well. As far as we know, this is the first study to corroborate the relationships between ‘Fuerte’ and these three green-skinned cultivars, reinforcing the strength of our approach.

Avocado races are distinguished by their origin [21], traits [7], and their morphological characteristics [3]. Because avocado races were determined, among other factors, by their putative geographical origins [3], one would expect to find a tight correlation between the place of collection of the deduced core-set accessions, and their proposed center of origin. Examination of the geographical origin of the Guatemalan core-set accessions indicated that, indeed, all nine accessions were collected by Ben- Ya’acov [19] from the Chiapas and San Marcos area, located at the highland region of Guatemala. By contrast, only four of seven Mexican core-set accessions were collected from different states in Mexico, whereas the 14 deduced West Indian core-set accessions were collected from various geographical regions (see Additional file 1). These biases might be related to human intervention, carried out for breeding purposes. In this context, it worth mentioning, that West Indian rootstocks especially selected for high tolerance to salinity and soil diseases, have been dispersed throughout the world [23]. It is therefore likely that the Mexican and West Indian core-set accessions were not necessarily collected from their genuine centers of origin.

It would be interesting to reconstruct the historical genealogy of the avocado cultivars from the genetic data. The analysis of IBD may assist in downstream parentage analysis with no a-priori knowledge about the genetic relationship of the accessions in the germplasm collection. In such case, the group of accessions related at a second-degree level can be used as candidates for a survey of putative-parents. This study may also contribute to preservation of the genetic diversity of *P. americana* and its sub-species races by sampling from each core set and from each type of hybrid as suggested by Guzmán et al. [24] upon composing a core-collection.

## Conclusions

Commonly, marker discovery is performed by genotyping the population under study and using the polymorphic loci. Our study proposed to discover, as a first step, the polymorphic loci. This step was done using resequencing pooled germplasm accessions from the collection followed by their alignment to a reference transcriptome to reduce the genome complexity. Consequently, a stock of genome-wide polymorphic loci was compiled, for future use. In a case that divergence to sub-species of races exist, sub-pooling of each race is required to maximize the prediction power of polymorphic loci. This study proposed that pooling the races based on botanical characterization solely might be misleading. Therefore, a core set of pure crop sub-species was identified, which can be used as a reference for race classification. This approach can serve as a framework in marker-assisted breeding. By using this framework, we revisit the accrued knowledge of avocado cultivars using a subset of discovered loci. While this core set and the knowledge about the kinship among avocado cultivars is useful for the avocado community, the framework can be applied to any crop.

## Methods

### Avocado accessions

Accessions in the Israeli avocado germplasm bank were classified according to the different botanical race groups based on key external characteristics [19]. This study included 100 accessions comprising the three avocado races: Guatemalan (17), Mexican (10) and West Indian (23) from the IAGB, 18 unidentified accessions, and 32 cultivar accessions, which some were developed by the Agricultural Research Organization (ARO) avocado breeding program and some are known commercial cultivars (Additional file 1: Table S1).

### Genomic DNA extraction and sequencing

gDNA was isolated from young avocado leaves grounded in liquid nitrogen; 2 g DNA, was extracted in 15 ml of extraction buffer (100 M Tris, pH 8.0, 1.5 M NaCl, 3% *w*/*v* CTAB, PVP, 1% *v*/v ß-mercaptoethanol) and 15 ml of chloroform: isoamyl alcohol. After a second extraction in (chloroform:isoamyl alcohol 24:1), the DNA was precipitated in Ethanol, treated with 1 μl of 10 μg/ml ribonuclease A (Sigma), precipitated and re-suspended in water. The DNA was quantified in a NanoDrop spectrometer and by separation on 0.8% agarose gels. Pure DNA sample aliquots of 0.5 μg were pooled by mixing the extracted DNA in equal amounts. DNA samples were pooled based on their botanical classification as either West Indian, Guatemalan, or Mexican.

The pools of gDNA were sequenced at the High-Throughput Sequencing and Genotyping Unit**,** Roy J Craver Biotechnology Center, University of Illinois on the Illumina HiSeq-2000 platform, which resulted in a yield of 30 Gb each. Consequently, the expected coverage is 33X, based on the estimation of genome size as 900 Mb. Raw data were uploaded to Sequence Reads Archive (SRA) accession no.: PRJNA438468.

### SNP discovery

Reads were improved by trimming the low quality 3′ ends using Sickle [25] with a quality threshold of 30. The remaining reads were aligned to the avocado reference transcriptome [26] using bowtie2 [27]. A preliminary study suggested that very high coverage turns into false positives when were validated on the collection’s accessions (unpublished data). Therefore, the SNP screening was restricted by a lower bound (depth = 6 reads) and upper bound (depth = 200 reads) around the peak of the coverage distribution (Additional file 4; Figure S1). SNPs were discovered by running the VarScan [28] program on the mpileup format of samtools [29] as input, resulting in a table of SNPs and the proportion of alleles for each pool (Additional file 3).

### Genotype calls

Specific primers for the SNPs were designed based on the flanking region from the avocado unigenes. The assays were run according to manufacture instructions on an EP1 platform using 96 × 96 chips following standard Fluidigm protocols (http://www.fluidigm.com) with a minor modification of four no template controls (NTC) samples instead of one. The SNP assays were used to screen the 100 accessions’ DNA samples by running on ‘FR96.96′ arrays of the EP1 Fluidigm platform according to the manufacturer’s instructions.

### Filtering and validation

To guarantee loci with true positive SNPs, only SNPs with explicitly separated clusters were chosen from the Fluidigm software for genotype calling. SNP’s loci with > 10% no calls and samples with > 30% no calls were filtered out. Minor allele frequency SNPs with PIC< 0.1 and linked loci with r^2^ > 0.7 were filtered out as well, leaving 109 SNPs out of the randomly selected 192. The polymorphism information content was calculated as

$$ PIC=1-\sum \limits_i^n{P}_i^2 $$

where i is the i^th^ allele [30]. The linkage disequilibrium of two SNPs at two loci was calculated as

$$ {r}^2=\frac{p_{11}\ast {p}_{22}-{p}_{12}\ast {p}_{21}}{\sqrt{p_1\ast {p}_2\ast {q}_1\ast {q}_2}} $$

where *p*_1_ and *p*_2_ are the proportions of alleles 1 and 2, respectively, in one locus and *q*_1_ and *q*_2_ are the proportions of alleles 1 and 2, respectively, in another locus. *p*_11_, *p*_12_, *p*_21_, *p*_22_ are the observed proportions of the four possible haplotypes from the two loci.

Predicted positive calls were defined as loci that were polymorphic in the pooled sequences and predicted negative calls were any loci that were not called as polymorphic. Actual positive and negative calls were the calls from the genotyping of individuals, as determined using Fluidigm. The true positive rate was the proportion of true positives (the intersection of the predicted positive calls and the actual positive calls) from the total actual positive calls.

### Genetic structure and relationship

To assess the relationship between pairs of avocado trees, we estimated the genetic distance as D = [1-proportion of shared alleles (PSA)]. PSA was calculated as:

$$ PSA=\frac{\sum_{i=1}^L{PS}_i}{2\ast L} $$

where PS is the proportion of shared alleles for each locus and L is the total number of loci [31].

Hierarchical clustering was performed on a pairwise D distance matrix and the Ward’s agglomerative method [32] was applied. The confidence limits of the tree topology were calculated by applying the bootstrap method (1000 resampling of loci). To count the number of bipartitions that fit the tree we used the ‘ape’ R-package [33, 34] and presented the bootstrap values.

The subpopulation structure of the germplasm collection was estimated by running a simulation of STRUCTURE software v2.3.3 [35] with 5000 burn-in periods and 50,000 repetitions. The number of populations, K, was inferred by running the simulation of K = 1 to K = 10 (20 runs for each K) and using the likelihood method of ΔK [36]. In the column graph a specific K was the summation of the different solutions of the 20 runs using CLUMPAK [37]. To classify the accessions relative to the core-race accessions we used STRUCTURE with prior population information, where the core samples where used to define the three ancestral sub-populations. Here too, we ran 20 simulations and integrated them by averaging over the ancestral proportion of each sample.

The fixation index F_ST_ [38] was calculated as:

$$ {F}_{ST}=\frac{H_T-{H}_S}{H_T} $$

where F_ST_ is the genetic differentiation of a subpopulation due to genetic drift, H_S_ is the weighted average of all subpopulations’ expected heterozygosity, and H_T_ is the expected heterozygosity in the entire population (germplasm collection).

### Kinship analysis and principal component analysis (PCA)

Principal component analysis was performed on the core samples only using SNPRelate R-package [39] snpgdsPCA() function. We then obtained the SNP loadings (or SNP eigenvectors) using the snpgdsPCASNPLoading() function. We projected all individuals onto the existing space of the principal components with the snpgdsPCASampLoading() function.

The kinship between pairs of avocado accessions was estimated using the robust method of moments called KING, which calculates moments of IBD coefficients [40]. The accessions that were related to each other by at most a second-degree relationship (i.e., parent-child, siblings, and grandparent-grandchild) estimated using SNPRelate R-package and were connected by edges in the PCA plot.

## Additional files

Additional file 1: **Table S1.** The Agricultural Research Organization (ARO) avocado collection. (DOCX 34 kb)

Additional file 2: **Table S2.** Classification of avocado accessions. (DOCX 26 kb)

Additional file 3: **Table S3.** A list of single nucleotide polymorphisms (SNPs) in avocado transcriptome. **Header descriptions**: Contig – mRNA contig ID; Position – SNP position; Ref allele – Allele call on the reference contig; Alt allele – Allele call which is different from the reference allele; Guatemalan coverage – Reads’ depth of the Guatemalan pool sequencing; Guatemalan alt prop – proportion of the alternative allele; Mexican coverage – Reads’ depth of the Mexican pool sequencing; Mexican coverage – Reads’ depth of the Mexican pool sequencing; WestIndian coverage – Reads’ depth of the WestIndian pool sequencing; WestIndian alt prop – proportion of the alternative allele; Guatemalan – Guatemalan polymorphism call; Mexican – Mexican polymorphism call; WestIndian – WestIndian polymorphism call; Contig sequence – Sequence of the contig; **Polymorphism call description**: AA – homozygous to reference allele; BB – homozygous to alternative allele; AB - heterozygous (XLSX 50424 kb)

Additional file 4: **Figure S1.** Distribution of avocado transcriptome coverage. Three pools of genomic DNA representing the three avocado races were sequenced on Illumina Hi-Seq platform. Reads were aligned with the avocado transcriptome after trimming adpters and cliping of low quality base calls. The distributions of read depths for the three pools are illustrated as solid line (Guatemalan pool), dotted line (Mexican pool), and dashed line (West Indian pool). Red vertical lines are the bounds the coverage for SNP discovery. (DOCX 183 kb)

Additional file 5 **Figure S2.** Correlation between the allele frequency estimated from the pools and that from genotype calling of the collection’s individuals. The proportion of SNP alleles were calculated for each locus and each pool. The proportions of SNP alleles of the corresponding loci and avocado accessions were calculated from the genotype calls using the Fluidigm platform. (DOCX 112 kb)

## Abbreviations

BNGA: National avocado germplasm depository
GBS: Genotype-by-sequencing
IAGB: Israeli avocado germplasm bank
IBD: Identity by descent
ISSR: Inter-simple sequence repeat
LOH: Loss-of-heterozygosity
NGS: Next-generation sequencing
PCA: Principal component analysis
RAPD: Random amplified polymorphic DNA
RFLP: Restriction fragment length polymorphism
SNP: Single-nucleotide polymorphisms
