# Supplementary material for: Genetic diversity of avocado (Persea americana Mill.) germplasm using pooled sequencing
Source: BMC Genomics. 2019 May 15;20:379. doi: 10.1186/s12864-019-5672-7 (PMC6521498; doi:10.1186/s12864-019-5672-7)
Supplement: Supplementary file 1 — Table S1. The Agricultural Research Organization (ARO) avocado collection. (DOCX 34 kb) [file 12864_2019_5672_MOESM1_ESM.docx]

**Genetic diversity of avocado (*Persea americana* Mill.) germplasm using pooled sequencing.**

Supplementary materials

**Table S1**. The Agricultural Research Organization (ARO) avocado collection.

| Sample name | Accession | Country of origin | Category | Reference |
| --- | --- | --- | --- | --- |
| Avo-84 | El Pino | Honduras | West-Indian | [1] |
| Avo-16 | Rirotonga A | Malaysia | West-Indian | [1] |
| Avo-62 | Hojancha 2 | Costa Rica | West-Indian | [1] |
| Avo-1 | Miremar | Costa Rica | West-Indian | [1] |
| Avo-78 | Argui 1 | Canary Island | West-Indian | [1] |
| Avo-15 | Gordienko 1 | Costa Rica | West-Indian | [1] |
| Avo-8 | Puerto Jimenez | Costa Rica | West-Indian | [1] |
| Avo-47 | Huixtla | Mexico | West-Indian | [1] |
| Avo-2 | Tela 2 | Honduras | West-Indian | [1] |
| Avo-30 | Amatlan | Mexico | West-Indian | [1] |
| Avo-75 | S. Javier 8 | Canary Island | West-Indian | [1] |
| Avo-18 | Tantima 2 | Mexico | West-Indian | [1] |
| Avo-3 | Tapachula | Mexico | West-Indian | [1] |
| Avo-6 | San Sebastian11 | Mexico | West-Indian | [1] |
| Avo-82 | Arbol 1 | Panama | West-Indian | [1] |
| Avo-74 | S. Javier 1 | Canary Island | West-Indian | [1] |
| Avo-19 | Orizaba 3 | Mexico | West-Indian | [1] |
| Avo-11 | Avocatosa 2 | Mexico | West-Indian | [1] |
| Avo-4 | Tela 3 | Honduras | West-Indian | [1] |
| Avo-20 | Frowe | USA | West-Indian | [1] |
| Avo-12 | Guzman | Mexico | West-Indian | [1] |
| Avo-58 | Urdesa S | Ecuador | West-Indian | [1] |
| Avo-160 | Antigua | Mexico | West-Indian | [1] |
| Avo-32 | P.i.s. 6915 | USA | Unidentified | [1] |
| Avo-77 | El Cercado | Spain | Unidentified | [1] |
| Avo-46 | Irigaray 141 | Costa Rica | Unidentified | [1] |
| Avo-85 | Aguacate de monte | Costa Rica | Unidentified | [1] |
| Avo-33 | Maskaria 1 | Ecuador | Unidentified | [1] |
| Avo-76 | La Piscina | Spain | Unidentified | [1] |
| Avo-61 | Los Angeles 3 | Costa Rica | Unidentified | [1] |
| Avo-45 | Irigaray 148 | Costa Rica | Unidentified | [1] |
| Avo-86 | Fredi 5 | Costa Rica | Unidentified | [1] |
| Avo-22 | P. americana. H1-72 | Honduras | Unidentified | [1] |
| Avo-56 | Marichal | Costa Rica | Unidentified | [1] |
| Avo-26 | Apakia 1 | Ecuador | Unidentified | [1] |
| Avo-87 | M. Pedro 2 |  | Unidentified | [1] |
| Avo-80 | El Charco 17 | Spain | Unidentified | [1] |
| Avo-13 | El Venado | Mexico | Unidentified | [1] |
| Avo-35 | Guat 1 | Ecuador | Unidentified | [1] |
| Avo-59 | Guacimal | Costa Rica | Unidentified | [1] |
| Avo-81 | El Charco 1 | Spain | Unidentified | [1] |
| Avo-54 | Basaldua | Mexico | Mexican | [1] |
| Avo-38 | Km 43 | Mexico | Mexican | [1] |
| Avo-55 | Aguacatitlan | Mexico | Mexican | [1] |
| Avo-53 | Tezuitlan | Mexico | Mexican | [1] |
| Avo-60 | Tochomilco 1 | Mexico | Mexican | [1] |
| Avo-52 | Guayabamba | Ecuador | Mexican | [1] |
| Avo-57 | Rio Negro 1 | Ecuador | Mexican | [1] |
| Avo-49 | Aquila 2 | Mexico | Mexican | [1] |
| Avo-51 | Gainsville | USA | Mexican | [1] |
| Avo-50 | Banos | Ecuador | Mexican | [1] |
| Avo-39 | Sholola 1 | Mexico | Guatemalan | [1] |
| Avo-24 | San Marcos 1 | Guatemala | Guatemalan | [1] |
| Avo-31 | Comitan 3 | Mexico | Guatemalan | [1] |
| Avo-23 | Puerto Viejo | Costa Rica | Guatemalan | [1] |
| Avo-40 | Comitan 1 | Mexico | Guatemalan | [1] |
| Avo-37 | Amatenango | Mexico | Guatemalan | [1] |
| Avo-79 | Egami | USA | Guatemalan | [1] |
| Avo-41 | Nochan 3 | Guatemala | Guatemalan | [1] |
| Avo-83 | San Rafael | Guatemala | Guatemalan | [1] |
| Avo-44 | San Marcos 2 | Guatemala | Guatemalan | [1] |
| Avo-29 | Cuevas | Mexico | Guatemalan | [1] |
| Avo-21 | Rollie | USA | Guatemalan | [1] |
| Avo-42 | S.Cristobal Mer. 35 | Mexico | Guatemalan | [1] |
| Avo-43 | S.Cristobal Mer. 36 | Mexico | Guatemalan | [1] |
| Avo-88 | Aguacate de Anis | Honduras | Guatemalan | [1] |
| Avo-36 | Sholola 6 | Mexico | Guatemalan | [1] |
| Avo-28 | Palestina | Guatemala | Guatemalan | [1] |
| Avo-92 | Hass | California, USA | Cultivar | Guatemalan [2]; Mexican X Guatemalan [3]; Guatemalan [4]; Mexican X Guatemalan [5] |
| Avo-69 | Adi | Israel | Cultivar | Guatemalan [2]; Mexican X Guatemalan [6] |
| Avo-71 | Ettinger | Israel | Cultivar | Mexican X Guatemalan[2]; Mexican X Guatemalan [4] |
| Avo-66 | Bar | Israel | Cultivar | Mexican X Guatemalan [7] |
| Avo-73 | Reed | California, USA | Cultivar | Guatemalan [2]; Guatemalan [5] |
| Avo-65 | Moti | Israel | Cultivar | Guatemalan [7] |
| Avo-131 | Naor | Israel | Cultivar | Mexican X Guatemalan [7] |
| Avo-124 | Pinkerton | California, USA | Cultivar | Mexican X Guatemalan [2]; Mexican X Guatemalan [4]; Guatemalan (Chen et al., 2009) |
| Avo-108 | Iriet | Israel | Cultivar | Guatemalan [2]; Mexican X Guatemalan [8] |
| Avo-154 | Lavi | Israel | Cultivar | Mexican X Guatemalan [7] |
| Avo-90 | Edranol | California, USA | Cultivar | Guatemalan [2] |
| Avo-123 | Fuerte | Mexico | Cultivar | Mexican X Guatemalan (Lahav and Gazit, 1994); Mexican X Guatemalan (Ashworth and Clegg, 2003); Mexican (Chen et al., 2009) |
| Avo-155 | Gem | California, USA | Cultivar | Mexican X Guatemalan (Ashworth and Clegg, 2003) |
| Avo-89 | Zutano | California, USA | Cultivar | Mexican X Guatemalan (Lahav and Gazit, 1994); Mexican (Ashworth and Clegg., 2003); Mexican X Guatemalan (Chen et al., 2009) |
| Avo-125 | Ardith | California, USA | Cultivar | Guatemalan X Mexican (Lahav and Gazit, 1994) |
| Avo-145 | Galil | Israel | Cultivar | Mexican [8] |
| Avo-129 | Red Label | California, USA | Cultivar |  |
| Avo-114 | Horshim | Israel | Cultivar | Mexican X Guatemalan [2] |
| Avo-99 | Withsell | California, USA | Cultivar | Guatemalan [2]; Mexican X Guatemalan [5] |
| Avo-140 | Noble (BL667) | California, USA | Cultivar | Mexican X Guatemalan [4] |
| Avo-159 | Arad | Israel | Cultivar | Mexican X Guatemalan [2]; Mexican X Guatemalan [6] |
| Avo-102 | Teague | Israel | Cultivar | Mexican [2]; Mexican X Guatemalan [5] |
| Avo-121 | Bacon | California, USA | Cultivar | Mexican X Guatemalan [2]; Mexican X Guatemalan [4]; Guatemalan [5] |
| Avo-98 | Esther | California, USA | Cultivar | Guatemalan [2]; Guatemalan [5] |
| Avo-141 | N-151-2 | California, USA | Cultivar | Guatemalan [2] |
| Avo-143 | Gwen | California, USA | Cultivar | Guatemalan X Mexican [2]; Guatemalan X Mexican [4]; Guatemalan [5] |
| Avo-103 | Sharwil | Australia | Cultivar | Guatemalan [2] |
| Avo-96 | Wurtz | California, USA | Cultivar | Guatemalan X Mexican [2] |
| Avo-112 | Benik | Guatemala | Cultivar | Guatemalan [2] |
| Avo-104 | Shepard | Australia | Cultivar | Guatemalan (http://www.ucavo.ucr.edu) |
| Avo-97 | Lamb Hass (BL122) | California, USA | Cultivar | Mexican X Guatemalan [4] |
| Avo-142 | Oshri | Israel | Cultivar | Mexican [2] |

**References**

1. Ben-Ya’acov A, Zilberstaine M, Goren M, Tome E. The Israeli avocado germplasm bank: where and why the items had been collected. Proc V World Avocado Congr. 2003;:13–19.

2. Lahav E, Gazit S. World listing of avocado cultivars according to flowering type. Fruits Fr. 1994.

3. Schnell RJ, Brown JS, Olanno CT, Power EJ, Krol CA. Molecular characterization of avocado germplasm with a new set of SSR and EST-SSR markers: genetic diversity, population structure, and identification of race-specific markers in a group of cultivated genotypes. J Amer Soc Hort Sci. 2003;128(6):881–9.

4. Ashworth VETM, Clegg MT. Microsatellite Markers in Avocado (Persea americana Mill.): Genealogical Relationships Among Cultivated Avocado Genotypes. J Hered. 2003;94:407–15.

5. Chen H, Morrell PL, Ashworth VETM, Cruz M de la, Clegg MT. Tracing the Geographic Origins of Major Avocado Cultivars. J Hered. 2009;100:56–65.

6. Lahav E, Israeli T, Regev I, Ardity H, Lavi U, Chemo M, et al. “Arad” - A new avocado cultivar. Hortscience. 2005;40:488–488.

7. Regev I, Ardity H, Israeli T, Lahav E, Lavi U, Chemo M, et al. “Lavi” - A new avocado cultivar. Hortscience. 2005;40:489–489.

8. Lahav E, Lavi U, Zamet D, Degani C, Gazit S. IRIET - A NEW AVOCADO CULTIVAR. Hortscience. 1989;24:865–6.
