# Supplementary material for: Genetic diversity of avocado (Persea americana Mill.) germplasm using pooled sequencing
Source: BMC Genomics. 2019 May 15;20:379. doi: 10.1186/s12864-019-5672-7 (PMC6521498; doi:10.1186/s12864-019-5672-7)
Supplement: Supplementary file 2 — Table S2. Classification of avocado accessions. (DOCX 26 kb) [file 12864_2019_5672_MOESM2_ESM.docx]

**Genetic diversity of avocado (*Persea americana* Mill.) germplasm using pooled sequencing.**

Supplementary materials

**Table S2**. Classification of avocado accessions.

| Sample code | Accession | Core | Known classification in literature | New classification | Mexican | Guatemalan | West Indian |
| --- | --- | --- | --- | --- | --- | --- | --- |
| Avo-84 | El Pino | WI | WI | WI | 0 | 0 | 1 |
| Avo-62 | Hojancha 2 | WI | WI | WI | 0 | 0 | 1 |
| Avo-1 | Miremar | WI | WI | WI | 0 | 0 | 1 |
| Avo-47 | Huixtla | WI | WI | WI | 0 | 0 | 1 |
| Avo-2 | Tela 2 | WI | WI | WI | 0 | 0 | 1 |
| Avo-30 | Amatlan | WI | WI | WI | 0 | 0 | 1 |
| Avo-18 | Tantima 2 | WI | WI | WI | 0 | 0 | 1 |
| Avo-3 | Tapachula | WI | WI | WI | 0 | 0 | 1 |
| Avo-6 | San Sebastian11 | WI | WI | WI | 0 | 0 | 1 |
| Avo-19 | Orizaba 3 | WI | WI | WI | 0 | 0 | 1 |
| Avo-4 | Tela 3 | WI | WI | WI | 0 | 0 | 1 |
| Avo-12 | Guzman | WI | WI | WI | 0 | 0 | 1 |
| Avo-58 | Urdesa S | WI | WI | WI | 0 | 0 | 1 |
| Avo-160 | Antigua | WI | WI | WI | 0 | 0 | 1 |
| Avo-55 | Aguacatitlan | M | M | M | 1 | 0 | 0 |
| Avo-53 | Tezuitlan | M | M | M | 1 | 0 | 0 |
| Avo-60 | Tochomilco 1 | M | M | M | 1 | 0 | 0 |
| Avo-52 | Guayabamba | M | M | M | 1 | 0 | 0 |
| Avo-57 | Rio Negro 1 | M | M | M | 1 | 0 | 0 |
| Avo-49 | Aquila 2 | M | M | M | 1 | 0 | 0 |
| Avo-51 | Gainsville | M | M | M | 1 | 0 | 0 |
| Avo-31 | Comitan 3 | G | G | G | 0 | 1 | 0 |
| Avo-40 | Comitan 1 | G | G | G | 0 | 1 | 0 |
| Avo-37 | Amatenango | G | G | G | 0 | 1 | 0 |
| Avo-44 | San Marcos 2 | G | G | G | 0 | 1 | 0 |
| Avo-29 | Cuevas | G | G | G | 0 | 1 | 0 |
| Avo-42 | S.Cristobal Mer. 35 | G | G | G | 0 | 1 | 0 |
| Avo-43 | S.Cristobal Mer. 36 | G | G | G | 0 | 1 | 0 |
| Avo-36 | Sholola 6 | G | G | G | 0 | 1 | 0 |
| Avo-28 | Palestina | G | G | G | 0 | 1 | 0 |
| Avo-82 | Arbol 1 |  | WI | WI | 0.01 | 0.051 | 0.938 |
| Avo-8 | Puerto Jimenez |  | WI | M X WI | 0.873 | 0.007 | 0.12 |
| Avo-75 | S. Javier 8 |  | WI | M X WI | 0.641 | 0.024 | 0.335 |
| Avo-74 | S. Javier 1 |  | WI | M X WI | 0.548 | 0.016 | 0.436 |
| Avo-78 | Argui 1 |  | WI | M X G X WI | 0.243 | 0.236 | 0.521 |
| Avo-16 | Rirotonga A |  | WI | G X WI | 0.006 | 0.173 | 0.821 |
| Avo-15 | Gordienko 1 |  | WI | G X WI | 0.003 | 0.136 | 0.861 |
| Avo-20 | Frowe |  | WI | G X WI | 0.003 | 0.456 | 0.541 |
| Avo-11 | Avocatosa 2 |  | WI | G | 0.003 | 0.951 | 0.045 |
| Avo-32 | P.i.s. 6915 |  | UI | WI | 0.003 | 0.006 | 0.991 |
| Avo-77 | El Cercado |  | UI | WI | 0.002 | 0.002 | 0.996 |
| Avo-46 | Irigaray 141 |  | UI | WI | 0.005 | 0.01 | 0.985 |
| Avo-61 | Los Angeles 3 |  | UI | WI | 0.004 | 0.017 | 0.979 |
| Avo-45 | Irigaray 148 |  | UI | WI | 0.005 | 0.072 | 0.924 |
| Avo-56 | Marichal |  | UI | WI | 0.03 | 0.035 | 0.934 |
| Avo-80 | El Charco 17 |  | UI | WI | 0.003 | 0.005 | 0.992 |
| Avo-13 | El Venado |  | UI | WI | 0.017 | 0.009 | 0.974 |
| Avo-81 | El Charco 1 |  | UI | WI | 0.014 | 0.007 | 0.979 |
| Avo-33 | Maskaria 1 |  | UI | M X WI | 0.474 | 0.006 | 0.52 |
| Avo-26 | Apakia 1 |  | UI | M X WI | 0.539 | 0.004 | 0.456 |
| Avo-35 | Guat 1 |  | UI | M X WI | 0.447 | 0.007 | 0.546 |
| Avo-59 | Guacimal |  | UI | M X WI | 0.494 | 0.023 | 0.483 |
| Avo-87 | M. Pedro 2 |  | UI | M X G X WI | 0.238 | 0.238 | 0.524 |
| Avo-76 | La Piscina |  | UI | M | 0.958 | 0.01 | 0.032 |
| Avo-22 | P. americana. H1-72 |  | UI | M | 0.993 | 0.004 | 0.003 |
| Avo-85 | Aguacate de monte |  | UI | G X WI | 0.004 | 0.2 | 0.796 |
| Avo-86 | Fredi 5 |  | UI | G X WI | 0.013 | 0.349 | 0.638 |
| Avo-50 | Banos |  | M | M X WI | 0.784 | 0.005 | 0.211 |
| Avo-54 | Basaldua |  | M | M X G | 0.462 | 0.526 | 0.013 |
| Avo-38 | Km 43 |  | M | G X WI | 0.007 | 0.808 | 0.185 |
| Avo-23 | Puerto Viejo |  | G | WI | 0.002 | 0.004 | 0.993 |
| Avo-83 | San Rafael |  | G | WI | 0.005 | 0.093 | 0.902 |
| Avo-21 | Rollie |  | G | M X G | 0.718 | 0.271 | 0.011 |
| Avo-39 | Sholola 1 |  | G | M | 0.974 | 0.018 | 0.009 |
| Avo-24 | San Marcos 1 |  | G | G X WI | 0.013 | 0.685 | 0.302 |
| Avo-79 | Egami |  | G | G X WI | 0.006 | 0.654 | 0.34 |
| Avo-41 | Nochan 3 |  | G | G X WI | 0.003 | 0.47 | 0.527 |
| Avo-88 | Aguacate de Anis |  | G | G X WI | 0.049 | 0.192 | 0.759 |
| Avo-71 | Ettinger [C] |  | M X G | M X G | 0.453 | 0.544 | 0.004 |
| Avo-66 | Bar [C] |  | M X G | G | 0.104 | 0.893 | 0.003 |
| Avo-65 | Moti [C] |  | G | M X G | 0.132 | 0.821 | 0.048 |
| Avo-131 | Naor [C] |  | M X G | M X G | 0.118 | 0.872 | 0.01 |
| Avo-124 | Pinkerton [C] |  | M X G | M X G | 0.147 | 0.85 | 0.003 |
| Avo-138 | Edranol [C] |  | G | M X G | 0.519 | 0.477 | 0.004 |
| Avo-123 | Fuerte [C] |  | (M, M X G) | M X G | 0.366 | 0.631 | 0.004 |
| Avo-139 | Zutano [C] |  | (M, M X G) | M X G | 0.152 | 0.846 | 0.002 |
| Avo-125 | Ardith [C] |  | G | M X G | 0.214 | 0.783 | 0.003 |
| Avo-145 | Galil [C] |  | M | M X G | 0.614 | 0.382 | 0.004 |
| Avo-114 | Horshim [C] |  | M X G | M X G | 0.28 | 0.701 | 0.018 |
| Avo-159 | Arad [C] |  | M X G | M X G | 0.168 | 0.829 | 0.002 |
| Avo-102 | Teague [C] |  | (M, M X G) | M X G | 0.528 | 0.465 | 0.007 |
| Avo-121 | Bacon [C] |  | (G, M X G) | M X G | 0.504 | 0.493 | 0.003 |
| Avo-96 | Wurtz [C] |  | (G, M X G) | M X G | 0.201 | 0.793 | 0.006 |
| Avo-104 | Shepard [C] |  | G | M X G | 0.223 | 0.775 | 0.002 |
| Avo-142 | Oshri [C] |  | M | M X G | 0.694 | 0.3 | 0.006 |
| Avo-92 | Hass [C] |  | (G ,M X G) | G | 0.024 | 0.973 | 0.003 |
| Avo-69 | Adi [C] |  | M X G | G | 0.074 | 0.922 | 0.004 |
| Avo-73 | Reed [C] |  | G | G | 0.009 | 0.986 | 0.005 |
| Avo-108 | Iriet [C] |  | (G ,M X G) | G | 0.094 | 0.902 | 0.004 |
| Avo-154 | Lavi [C] |  | M X G | G | 0.078 | 0.918 | 0.003 |
| Avo-155 | Gem [C] |  | M X G | G | 0.015 | 0.982 | 0.003 |
| Avo-129 | Red Label [C] |  | UI | G | 0.004 | 0.993 | 0.003 |
| Avo-99 | Withsell [C] |  | G | G | 0.01 | 0.985 | 0.005 |
| Avo-140 | Noble (BL667) [C] |  | M X G | G | 0.014 | 0.983 | 0.003 |
| Avo-98 | Esther [C] |  | G | G | 0.047 | 0.95 | 0.003 |
| Avo-141 | N-151-2 [C] |  | G | G | 0.068 | 0.929 | 0.003 |
| Avo-127 | Gwen [C] |  | (G ,M X G) | G | 0.026 | 0.971 | 0.004 |
| Avo-103 | Sharwil [C] |  | G | G | 0.1 | 0.871 | 0.03 |
| Avo-112 | Benik [C] |  | G | G | 0.008 | 0.925 | 0.067 |
| Avo-97 | Lamb Hass (BL122) [C] |  | M X G | G | 0.008 | 0.987 | 0.004 |

WI – West Indian; G – Guatemalan; M – Mexican; [C] – cultivar; UI – unidentified.
