# Supplementary material for: Genetic diversity of avocado (Persea americana Mill.) germplasm using pooled sequencing
Source: BMC Genomics. 2019 May 15;20:379. doi: 10.1186/s12864-019-5672-7 (PMC6521498; doi:10.1186/s12864-019-5672-7)
Supplement: Supplementary file 4 — Figure S1. Distribution of avocado transcriptome coverage. Three pools of genomic DNA representing the three avocado races were sequenced on Illumina Hi-Seq platform. Reads were aligned with the avocado transcriptome after trimming adpters and cliping of low quality base calls. The distributions of read depths for the three pools are illustrated as solid line (Guatemalan pool), dotted line (Mexican pool), and dashed line (West Indian pool). Red vertical lines are the bounds the coverage for SNP discovery. (DOCX 183 kb) [file 12864_2019_5672_MOESM4_ESM.docx]

**Genetic diversity of avocado (*Persea americana* Mill.) germplasm using pooled sequencing.**

Supplementary materials


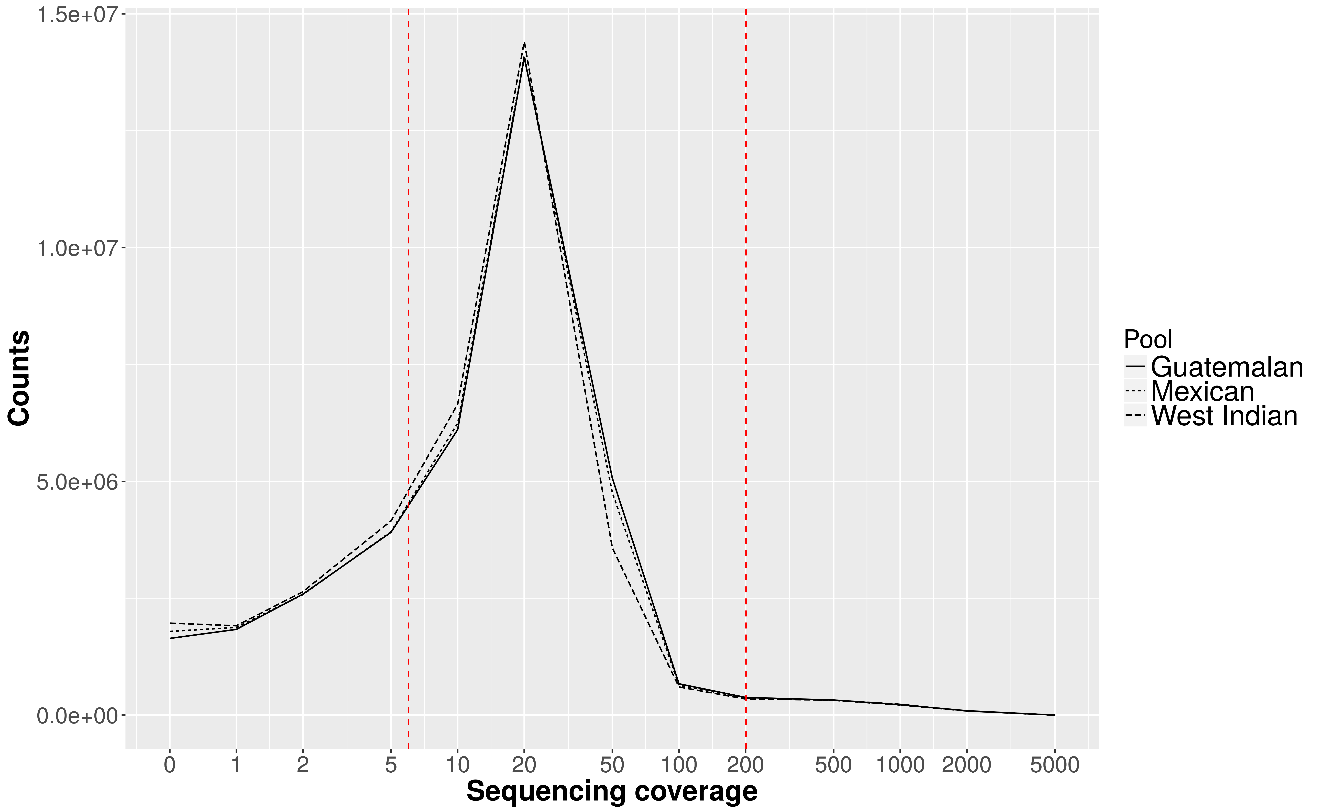


**Figure S1.** Distribution of avocado transcriptome coverage. Three pools of genomic DNA representing the three avocado races were sequenced on Illumina Hi-Seq platform. Reads were aligned with the avocado transcriptome after trimming adpters and cliping of low quality base calls. The distributions of read depths for the three pools are illustrated as solid line (Guatemalan pool), dotted line (Mexican pool) , and dashed line (West Indian pool). Red vertical lines are the bounds the coverage for SNP discovery.
