# Supplementary material for: Genetic diversity of avocado (Persea americana Mill.) germplasm using pooled sequencing
Source: BMC Genomics. 2019 May 15;20:379. doi: 10.1186/s12864-019-5672-7 (PMC6521498; doi:10.1186/s12864-019-5672-7)
Supplement: Supplementary file 5 — Figure S2. Correlation between the allele frequency estimated from the pools and that from genotype calling of the collection’s individuals. The proportion of SNP alleles were calculated for each locus and each pool. The proportions of SNP alleles of the corresponding loci and avocado accessions were calculated from the genotype calls using the Fluidigm platform. (DOCX 112 kb) [file 12864_2019_5672_MOESM5_ESM.docx]

**Genetic diversity of avocado (*Persea americana* Mill.) germplasm using pooled sequencing.**

Supplementary materials


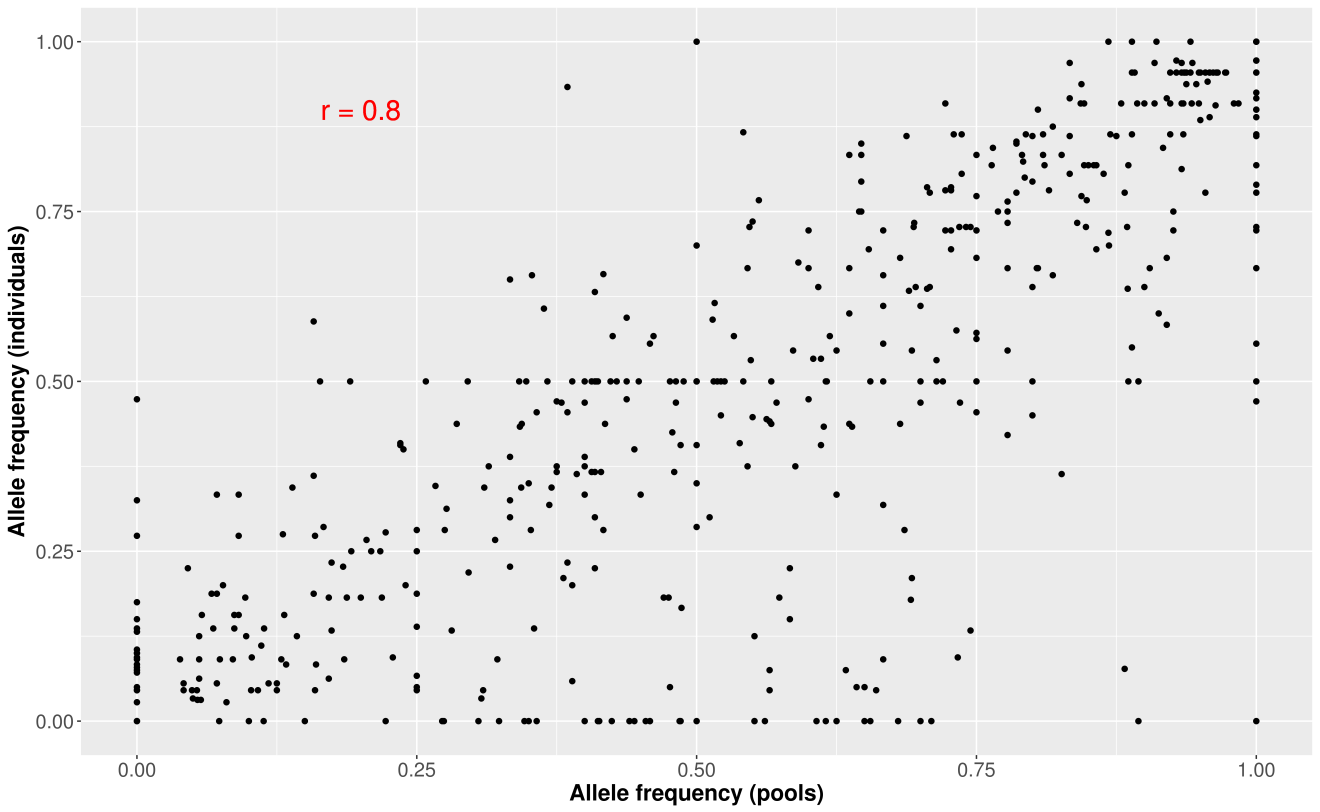


**Figure S2.** Correlation between the allele frequency estimated from the pools and that from genotype calling of the collection’s individuals. The proportion of SNP alleles were calculated for each locus and each pool. The proportions of SNP alleles of the corresponding loci and avocado accessions were calculated from the genotype calls using the Fluidigm platform.
